# Performance Study of Nickel Oxide Graphite Felts as Electrode Materials for Ferrochromium Flow Batteries

**DOI:** 10.1002/open.202500405

**Published:** 2025-09-02

**Authors:** Jia‐ning Xie, Xu Dai, Meng‐yao Liu, Xuan‐sen Li, Rui‐xian Feng, Hai‐lin Ren, Hao‐wen Wang, Xiao‐min Wang

**Affiliations:** ^1^ Xinjiang Key Laboratory of Novel Functional Materials Chemistry College of Chemistry and Environmental Science Kashi University Kashi Xinjiang 844000 P. R. China

**Keywords:** density functional theory, flow batteries, graphite felts, nickel oxide

## Abstract

Herein, the performance of nickel‐oxide‐modified graphite felts as electrode materials for Fe/Cr liquid flow batteries is investigated by combining density functional theory and experiments. The results show that the adsorption of NiO on Fe/Cr ions is more stable than that on graphite felt, and more electrochemically active sites are provided. The charge transfer after adsorption is larger, which is more conducive to the redox reaction during the charging and discharging processes. Finally, the experimental results further prove that the first discharge capacity of the nickel oxide‐modified graphite felt reaches 22.0 Ah L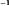, which is much higher than that of the unmodified graphite felt (12.4 Ah L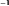). In addition, its energy efficiency could reach up to 59.5%, which is 27.3% higher than that of the original electrode.

## Graphical abstract

Differential charge density of graphite felt and nickel oxide and differential charge density of Fe and Cr adsorbed on graphite felt and nickel oxide. The dangling bonds of surface oxygen atoms in NiO can form bonding orbitals with Fe/Cr ions, achieving a maximum adsorption energy of 2.04 eV·Å^−^

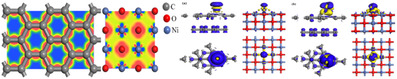

## 1 Introduction

In the current era, with the rapid advancement of science and technology and the continuous improvement of people's living standards, the demandfor energy has also surged. Traditional fossil energy sources such as oil and coal are no longer sufficient to meet people's daily needs and produce large amounts of greenhouse gases when burned, disturbing the natural balance and causing irreversible environmental damage. A large number of countries and regions around the world have formally proposed or committed to achieve the goal of “carbon neutrality,” and in the promotion of green chemistry, energy saving, and emission reduction, the energy revolution is imminent. The green transformation of industry and green technological innovation urgently need to develop wind and solar energy and other green, renewable, and clean energy.^[^ ^1^ ^,^ ^2^ ^]^ The development of renewable energy is a key path to mitigating climate change. However, clean energy such as solar energy is intermittent and unstable. For example, the intermittent supply characteristics of solar energy need to be coupled with energy storage systems to solve the problem of alternating day and night, and the energy provided by solar energy varies at different times of the day, so solar energy is usually not used as a baseload power source for electric power systems due to the lack of continuous output capacity. However, both daily human activities and societal development depend critically on a sustained energy supply. Under the path of deep decarbonization, energy storage technology is the key medium to solve the structural contradiction between the nondispatchability of clean energy and the continuous demand for energy on the demand side. Among various energy storage technologies, redox flow batteries (RFBs) are considered as one of the preferred technologies for large‐scale energy storage due to their long cycle life, high reliability, and low cost.^[^ ^3^ ^]^ The main constituent structures of ICRFB are electrodes, electrolyte, and ion‐exchange membrane, in which the electrodes have a key role in the battery system, providing a reaction site for the redox electric pair and determining the performance of the battery system.^[^ ^4^, ^5^ ^–^ ^6^ ^]^ After years of research on electrodes, it is now possible to prepare electrodes from a wide variety of raw materials (e.g., biocarbon materials, chemical wastes, etc.) and show very good results.^[^ ^7^ ^,^ ^8^ ^]^ However, graphite felt remains the most commonly used electrode material due to its stable electrochemical properties, large specific surface area (which facilitates redox reactions), and high porosity, which enhances electrolyte flow. Although graphite felts offer advantages such as high electrical conductivity and good stability, their poor wettability by electrolytes and limited electrochemical active sites increase the interfacial charge transfer impedance, thereby reducing the energy efficiency of the battery and hindering its practical application.^[^ ^9^ ^]^ In this regard, many scholars have modified graphite felts, and common methods include heat treatment to remove impurities covering the surface of graphite felts to produce grooves and increase the specific surface area; acid treatment; and introduction of metals or nonmetals.^[^ ^10^ ^,^ ^11^ ^]^ Yeonjoo Ahn et al. reported that nanoparticles embedded in graphite mats inhibited the hydrogen precipitation reaction and significantly improved the electrochemical activity of the redox pair of Cr ions.^[^ ^12^ ^]^ Chen et al. introduced silica into the surface of graphite felt to increase its activation point and added hydroxyl groups to improve its hydrophilicity by soaking the graphite felt in silicic acid for heat treatment and achieved an energy efficiency of 79.66% at a current density of 120 mAcm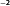.^[^ ^13^ ^]^ Su et al. effectively suppressed the hydrogen precipitation reaction and improved its redox reaction and charge transfer rate by modifying the graphite felt with In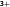.^[^ ^14^ ^]^ Jiang et al. doped B into graphite felt and used it as the electrode material for all‐vanadium liquid flow batteries compared to the pristine electrodes, which showed a significant increase in energy efficiency.^[^ ^15^ ^]^ The reversibility and electrochemical kinetics of graphite felt electrodes were improved by the modification of graphite felt electrodes with SnO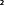 by Feng et al. The SnO
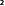
‐ modified graphite felt electrodes exhibited greater discharge capacity, higher electrolyte utilization, and lower polarization than blank cells.^[^ ^16^ ^]^ Subsequently, many metal oxide modified graphite felts, such as PbO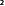, ZrO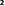, CoO, etc., have shown very excellent performance.^[^ ^17^, ^18^ ^–^ ^19^ ^]^ Based on this, this paper considers the preparation of nickel oxide‐modified graphite felts using alkaline nickel carbonate as a nickel source and explores their performance as electrode materials for ferrochromium flow batteries.^[^ ^9^ ^]^

## 2 Results and Discussion

### 2.1 Structure Analysis of Graphite Felt and Nickel Oxide

**Figure 1:**
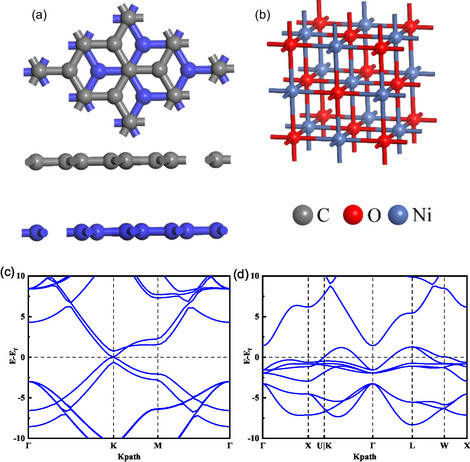
a) Schematic structure of graphite felt. b) Schematic structure of nickel oxide. c,d) Electronic energy band structure of graphite felt and nickel oxide.

The structures of graphite felts and nickel oxide are shown in **Figure** **1**a,b. The cubic phase NiO has a NaCl‐type face‐centered cubic structure, and the cell parameter is 4.16 Å and the bond length of Ni—O is 2.084 Å after sufficient atomic relaxation under the generalized gradient approximation (GGA) and Perdew–Burke–Ernzerhof (PBE) generalization, which is very close to experimental results.^[^ ^20^, ^21^ ^–^ ^22^ ^]^ Similarly, the graphite felt has a lattice parameter of 2.45 Å and a C—C bond length of 1.419 Å, which is also in good agreement with the experimental results.^[^ ^23^ ^]^ Figure 1c,d demonstrate the electronic energy band structure diagrams of graphite felt and nickel oxide, where the highest energy level occupied by the electrons of the system is the Fermi energy level, that is, Ef. Even though the Fermi energy level crosses a larger number of energy bands in the energy band structure of NiO, this is due to its own properties; Ni atoms have magnetic properties, and the number of electrons spinning up and down in the system does not coincide with the number of electrons in the system, leading to the interaction of electrons splitting near the Fermi energy level into more energy levels. It can be clearly seen that the VBM is located at the L point and the CBM is located at the Γ point,^[^ ^24^ ^]^ which indicates that NiO belongs to a typical indirect bandgap semiconductor, and the Fermi energy level is located in the valence band of the p‐type semiconductor. A very remarkable feature of the energy band structure of graphite is the presence of a Dirac cone at the K point, which is composed of the pz orbital electrons of C, and is closely related to the various unique properties of graphite as well as the high carrier mobility.^[^ ^25^ ^]^

### 2.2 Application of Nickel Oxide in Electrode Reactions

**Figure 2:**
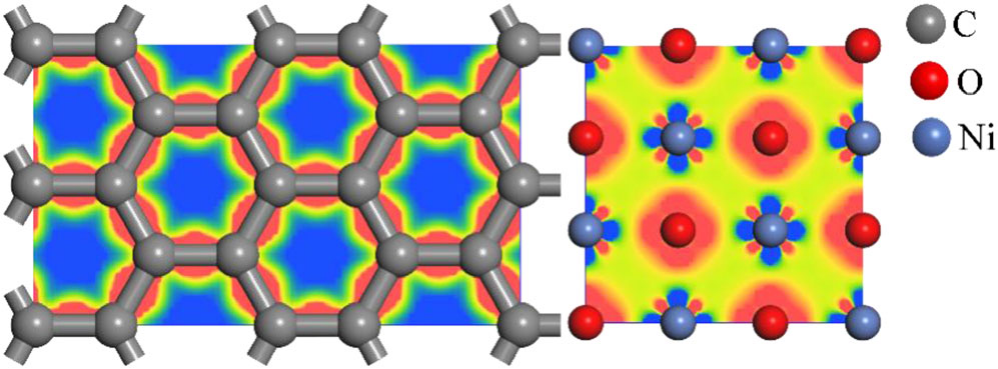
Differential charge density of graphite felt and nickel oxide.

**Figure 3:**
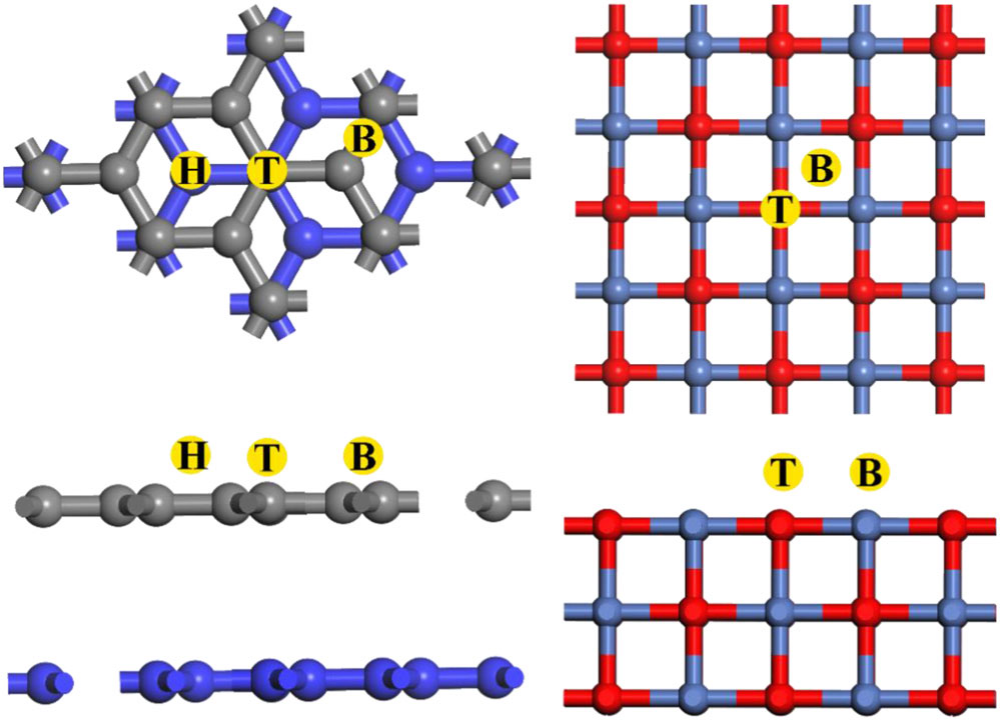
Possible adsorption sites of Fe/Cr ions on graphite felt and nickel oxide surfaces.

In order to determine the adsorption positions of the 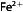/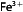 and 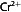/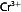 redox couples on the surface of NiO, the differential charge densities of graphite felt and nickel oxide were calculated, as shown in **Figure** **2**. In NiO, electrons are predominantly localized near oxygen atoms, whereas in graphite felt, they are primarily distributed between the C—C bonds. Based on this paper, it is believed that there are two main adsorption positions for NiO, which are the top position and the bridge position of O atoms; and there are three main adsorption positions for the graphite felt, which are the top position and the bridge position of C atoms as well as the center of the six‐membered ring of C, as shown in **Figure** **3**.^[^ ^26^ ^]^ After sufficient atomic relaxation, it was found that the adsorbed ions at the bridge site of NiO move to the top site, and both the top site and the bridge site in the graphite felt move to the center of the six‐membered ring of C, which suggests that only one stable adsorption site exists in both structures. The energy changes, adsorption heights, and charge transfers after adsorption completion for each structure are given in **Table** **1**. The following three conclusions can be drawn from the data in the table. (1) The adsorption stability of 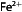/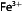 is higher than that of 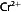/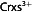 redox pairs for both nickel oxide and graphite felts; (2) the adsorption capacity of NiO is much larger than that of graphite felts for Fe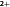/Fe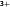 and Cr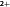/Cr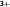 redox pairs, and it can provide more active sites during charging and discharging; and (3) the adsorption stability of 3‐valent ions is higher than 2‐valent ions for both Fe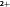/Fe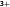 and Cr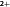/Cr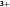, the adsorption stability of 3‐valent ions is higher than that of 2‐valent. In order to further understand this phenomenon, we carried out further calculations on the adsorption models of graphite felt and nickel oxide.

**Table 1:** Structural changes of Fe/Cr ions adsorbed on graphite mats and nickel oxide.

	Nickel oxide			Graphite felt		
	$E_{\text{ad}}$ [eV Å 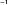 ]	h [Å]	Charge	$E_{\text{ad}}$ [eV Å 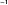 ]	h [Å]	Charge
Fe 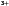	−2.04	1.67	0.563	−0.64	1.724	1.641
Fe 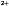	−0.97	1.60	0.442	−0.31	1.715	1.498
Cr 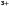	−1.83	1.68	0.387	−0.58	1.693	1.708
Cr 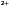	−0.92	1.64	0.252	−0.29	1.687	1.503

**Figure 4:**
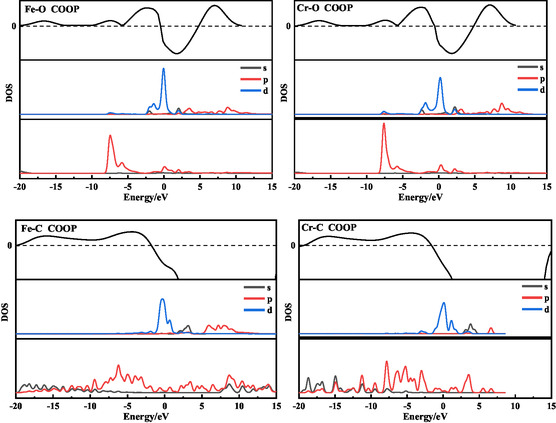
Crystal orbital layout and local density of states between Fe/Cr ions and O/C's.

**Figure 5:**
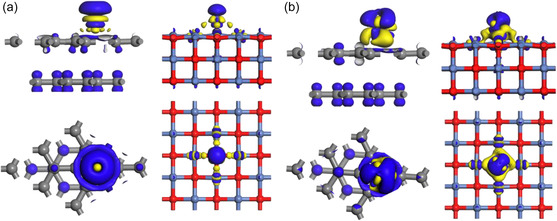
a) Differential charge density of Fe adsorbed on graphite felt and nickel oxide. b) Differential charge density of Cr adsorbed on graphite felt and nickel oxide.

**Figure** **4** shows the crystal orbital layout and local density of states of each adsorption model, it can be seen that when Fe/Cr is adsorbed on the surface of NiO, the dangling bonds of the O atoms on the surface can be easily combined with the empty orbitals of Fe/Cr, and the bonding orbitals are dominated by the formation of bonding orbitals down to the Fermi energy level, and at this time, the O will form covalent bonds with Fe/Cr. Mulliken bond population analysis also indicates O—Fe and O—Cr bond orders of 0.29 and 0.25 electrons, respectively. When Fe/Cr is adsorbed on the graphite felt surface, only a small number of 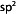 hybridized orbitals of C atoms will form bonding orbitals with the d
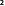
sp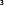 hybridized orbitals of Fe/Cr. Near the Fermi level, electrons in the carbon p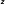 orbitals predominantly occupy antibonding states. It can also be seen from the differential charge density plot in **Figure** **5** that charge depletion is exhibited around the C atom, which is the transfer of electrons from the p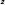 orbitals of C into the d
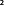
sp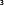 hybridization orbitals of Fe/Cr. At this time, the layout numbers of the C—Fe and C—Cr bonds are −0.17 and −0.19, respectively, which further indicates that the bonding conditions between C and Fe/Cr are not satisfied, and only Coulombic attraction can be relied upon.

The electrochemical reaction on the surface of graphite felt fibers during charging and discharging of ICRFB can be described by the Butler–Volmer equation as^[^ ^27^ ^,^ ^28^ ^]^

(1) $$j_{-}=\alpha_{-}Fk_{-}{\left(C_{\text{Fe(III)}}\right)}^{\alpha_{-}}{\left(C_{\text{Fe(II)}}\right)}^{\alpha_{+}}\left\{\exp\;\left(\frac{\alpha_{+}F\eta_{1}}{RT}\right)+\exp\;\left(\frac{\alpha_{-}F\eta_{1}}{RT}\right)\right\}$$

(2) $$j_{+}=\alpha_{+}Fk_{+}{\left(C_{\text{Cr(II)}}\right)}^{\alpha_{-}^{1}}{\left(C_{\text{Cr(III)}}\right)}^{\alpha_{+}^{1}}\left\{\exp\;\left(\frac{\alpha_{+}^{1}F\eta_{2}}{RT}\right)+\exp\;\left(\frac{\alpha_{-}^{1}F\eta_{2}}{RT}\right)\right\}$$

The 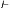 and $j_{+}$ scores are the local electric current densities at the anodic and polar electrodes, respectively, when the redox reaction occurs. Where $\alpha_{+}$/$\alpha_{-}$ min is the cathode and anode charge transfer coefficient, F is the Faraday constant, $k_{-}$/$k_{+}$ is the standard rate constant for the redox reaction at the anodic pole, $C_{\text{Fe(III)}}$/$C_{\text{Fe(II)}}$ is the concentration of each ion on the surface of the electrode, and $\eta_{1}$/$\eta_{2}$ are the activation overpotentials for redox reactions, respectively.^[^ ^29^ ^]^ According to the calculation results above, we can learn that NiO has more active sites and can spontaneously adsorb Fe/Cr ions in the solution, which increases the concentration of active ions on the electrode surface, that is, $C_{\left(i\right)}$ in the formula, and thus improves the local electric reaction current. And the charge transfer of Fe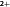/Fe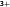 and Cr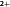/Cr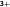 redox pairs on the NiO surface is higher, which is more favorable for the redox reaction, and in this regard, we can predict that the NiO‐modified graphite felts will have a more excellent performance in ICRFB.

**Figure 6:**
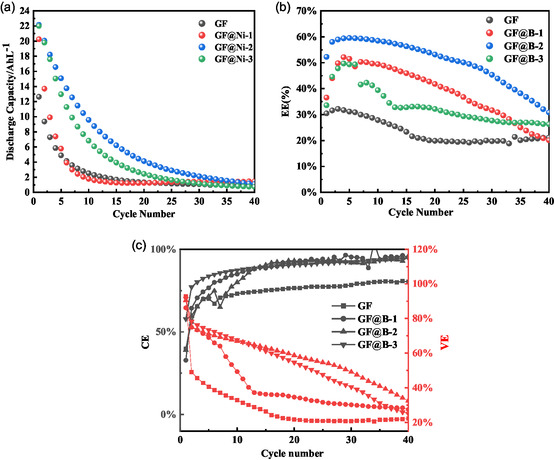
Single‐cell performance test. a) 40 charge/discharge cycling curves for each sample at a current density of 60 mAcm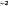. b) Energy efficiency of each sample during cycling. c) Voltage efficiency and Coulombic efficiency of each sample during cycling.

**Figure** **6**a shows the cycling stability of a single cell assembled from each graphite felt sample for 40 charges and discharges. The discharge capacity of all samples exhibited significant initial fading but gradually stabilized during cycling. The initial discharge capacities for GF, GF@Ni‐1, GF@Ni‐2, and GF@Ni‐3 are 12.4, 20.2, 22.0, and 22.1 Ah L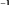, respectively. NiO‐modified GF demonstrates superior capacity due to strong Fe/Cr adsorption. Although the capacity of each sample decayed substantially after 40 cycles, the capacity of the two modified samples was consistently higher than that of GF throughout the cycling process, except for GF@Ni‐1, which had a lower NiO content. Combined with the change in energy efficiency (EE) of each sample during the cycling process, as shown in Figure 6b, it can be found that the graphite felts modified by NiO all show a tendency of increasing and then gradually decreasing, which indicates that NiO undergoes several activations in the charge/discharge cycle before reaching the optimal performance. The GF@Ni‐2 sample with the optimal performance achieved an energy efficiency of 59.5% after sufficient activation, while the pristine GF exhibited a maximum energy efficiency of only 32.2%, representing a nearly twofold improvement. Besides, according to the changes of the Coulomb efficiency (CE) and voltage efficiency (VE) during the cycling process of each sample in Figure 6c, it can also be found that the graphite mats modified with NiO have a large improvement in their CE and VE.

## 3 Conclusions

1) Density functional theory (DFT) calculations reveal that the electrons in the p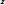 orbitals of carbon atoms in graphite felt form antibonding orbitals with the d
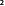
sp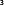 hybrid orbitals of Fe/Cr ions, thereby inhibiting the adsorption of Fe/Cr ions on the graphite felt surface. This results in low adsorption energy, where even the most active Fe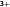 exhibits an adsorption energy of only 0.64 eV Å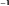. In contrast, the dangling bonds of surface oxygen atoms in NiO can form bonding orbitals with Fe/Cr ions, achieving a maximum adsorption energy of 2.04 eV Å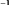. Additionally, the enhanced charge transfer further facilitates redox reactions during charge/discharge processes. 2) Experimental results demonstrate that the NiO‐modified graphite felt significantly improves capacity, achieving 22.0 Ah L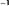, which is 7.6 Ah L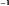 higher than that of pristine graphite felt. Moreover, over 40 charge/discharge cycles, the modified graphite felts exhibit superior performance in capacity retention, energy efficiency, Coulombic efficiency, and voltage efficiency compared to the unmodified counterpart.

## 4 Experimental Section

4.1

4.1.1

##### Calculation Method

All calculations in this article are based on DFT and are done using the Castep module on the Materials Studio software.^[^ ^30^ ^]^ The GGA and the PBE method are used to introduce an ultrasoft pseudopotential to calculate the exchange energy of all ions‐electrons.^[^ ^31^ ^]^ A TS dispersion correction is used to characterize the van der Waals effect at long distances. After a convergence test of the whole system (energy error less than 1 meV/atom), the plane‐wave up to energy is set to 700 eV, and the Brillouin zone is set up on a 3×3×1 Monkhorst packet grid. At the same time, to ensure sufficient accuracy, the restriction was relaxed for all atoms during the atomic relaxation phase until the self‐consistent field energy variation was less than 1×10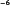 eV/atom, the total energy variation was less than 1×10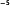 eV/atom, the maximum inter‐atomic stress was less than 0.03 eV/Å, and the maximum ionic step was less than 0.001 Å.^[^ ^32^ ^]^ To predict the stability of the structure, the adsorption energy 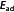 was calculated using the following equation^[^ ^33^ ^]^

(3) $$E_{\text{ad}}\;=\;(E_{\text{GF}‐M}-E_{\text{GF}}-\;EE_{\text{Fe}/\text{Cr}})/S$$

where *E*_GF‐M_ denotes the energy of the structure after adsorption of metal ions; *E*_GF_ denotes the energy of the structure without adsorption of metal ions; *E*_Fe/Cr_ denotes the energy of isolated Fe and Cr atoms; and *S* represents the area of the model.^[^ ^34^ ^]^

##### Sample Preparation

Take 5 mm graphite felt with deionized water to clean and remove impurities, dry and spare, recorded as GF. The GF was immersed in alkaline nickel carbonate solution containing 0.1 M, 0.2 M, and 0.3M for 24 h and then transferred to a tube furnace to be heated up to 600 °C at 10 °C min^−1^ under nitrogen atmosphere, and kept warm for 5 h. During the process, the alkaline nickel carbonate decomposed and bonded with the graphite felts, and the graphite felts were calcined with ultrasonic waves and dilute hydrochloric acid several times to remove the excess nickel hydroxide. After calcination, the excess nickel hydroxide was removed by washing several times with ultrasound and dilute hydrochloric acid, and the samples were labeled as GF@Ni‐1, GF@Ni‐2, and GF@Ni‐3.

## Conflict of Interest

The authors declare no conflict of interest.

## Author Contributions

**Jia‐ning Xie**, **Xu Dai, Meng‐yao Liu**, **Xuan‐sen Li**, **Rui‐xian Feng**, **Hai‐lin Ren**, **Hao‐wen Wang**, and **Xiao‐min Wang** contributed to the conceptualization, methodology, and execution of the research. **Xiao‐min Wang** (corresponding author) supervised the project, provided funding, and finalized the manuscript. All authors participated in data analysis and manuscript drafting and approved the final version.

## Data Availability

The data that support the findings of this study are available from the corresponding author upon reasonable request.
